# The Conference on Child Welfare

**Published:** 1921-07-16

**Authors:** 


### The Conference on Child Welfare.

It was unfortunate that National Baby Week, 1921, should have coincided with one of the most crowded weeks of an exceptionally crowded season. This circumstance may explain why it has attracted less publicity than its predecessors. So far as the inner circle was concerned, the voluntary and other workers to whom the movement to educate the public and to preserve child-life owes a great debt, the attendance at the Conferences held at the Central Hall from July 5 to 7 was numerous and interested, but the exhibition itself was less well attended, though there was much to learn from a visit. Clinics on Infant Consultations, Eyes, Teeth, and Breast-feeding were held daily, and a Mothers' Rest and Nursing Room and Nursery were available. Many babies, beautiful specimens of humanity, were present, one being carried about in a small hamper. Babies well and babies ill, infants and toddlers, the sighted and the blind, were provided for in the various exhibits, whilst demonstrations of cookery, laundry, and first aid were frequently given. A collapsible perambulator, invented by Miss F. C. Joseph, of Bridgwater, and easily made at home, was shown at the stall of the National Union of Trained Nurses, and a model day nursery at that of the National Society of Day Nurseries, whilst an interesting nursery school exhibit was that of the Gipsy Hill Training College. The skill of parents as a result of the teaching given at Infant Welfare Centres was shown in the articles displayed at the stall of the Association of Infant Welfare and Maternity Centres, which were made for the National Mothercraft competitions.

The Vital and Essential Forms of Child Welfare.

The programme of lectures of the second English-speaking Conference was detailed and comprehensive. Divided into three main sections—" Residential Provision for Mothers and Babies," " The Supply of Milk, its Physiological and Economic Aspects," and " Inheritance and Environment as Factors in Racial Health "—these lectures traversed a wide field, and were given by authorities on the various subjects. There were also professional lectures on " Common Infections in Mother and Child," by Dr. Eric Pritchard; " The Syphilitic Mother and her Infant," by Dr. John Adams; and " The Tuberculous Mother and her Infant," by Dr. Geoffrey Marshall. One of the most interesting discussions was that entitled " What are the most vital and essential forms of child-welfare work," introduced by Miss Zoe Puxley, of the Ministry of Health, and Professor H. Kenwood, M.O.H., Stoke Newington. The former urged the importance of co-operation between voluntary and official agencies, and suggested that the chief barriers to such co-ordination are difference in outlook and point of view, misunderstanding and suspicion, and prejudice. Very many voluntary workers think all officials are machine-made and without souls, whilst officials think voluntary workers are unbusinesslike and irregular. Allowing some truth for each point of view, the remedy is to humanise the official and make the voluntary worker businesslike, and there is plenty of opportunity for the achievement of both in child-welfare work. The presence of pioneer women in this work on the statutory committees is an excellent thing. Professor Kenwood, voicing the views of the administrative medical officers, laid stress on the educational aspect of the work, the transmission of knowledge both at the centres and in the homes of the people. The need of the hour is for more knowledge, not for more administrative powers. After education the next most important development is ante-natal work. No attempt should be made to measure the value of child-welfare centres in terms of the reduction of infant mortality; the value of the work done is in connection with survivals, to which it is very difficult to give statistical expression. Mrs. Hood (Women's Co-operative Guild), speaking as a working woman, urged as vital and essential factors (1) a better midwifery service; (2) a supply of home helps pending an adequate provision of maternity homes. Both midwifery and home helps to be State-aided as far as may be necessary to ensure an adequate supply. Other speakers who took part in the discussion were Miss Morson (Malvern), Dr. Dunstan Brewer (Swindon), Dr. Nash (Heston and Isleworth), Miss Brackenbury (Village Councils Federation), Mrs. Fowles (Birmingham), Miss Beaven (Liverpool), Miss Lee (Matlock Bath), and Dr. Harold Scurfield. Each made valuable contribution to the point at issue. Dr. Brewer believes that the problem is to get hold of the pregnant mother. Dr. Nash emphasised the importance of the work of the health visitor. The whole success of any scheme of child-welfare depends, he said, on her—she can make or wreck it. Miss Morson urged the importance of the toddlers as well as that of the mother, Mrs. Fowles the importance of ante-natal work. Miss Beaven wants more co-operation between the child's home and the out-patient department, and co-ordination by means of a Council of all people dealing with the child-welfare problem. If economies must be made by the Ministry the question of day nurseries might be considered; it is important that maternity and ante-natal work should not suffer. She dissented from the necessity for supplying free home helps; in her experience mothers would like to pay something themselves. Miss Lee (Matlock Bath) urged greater co-operation between midwives and the rural nurse associations, and between health visitors and midwives; this would make for economy and the more efficient visiting of ante-natal cases. The Queen's nurse-midwives should be recognised health visitors. Three vital and essential forms of child-welfare work, as stated by Dr. Harold Scurfield, are to prevent rickets, to improve the teeth, and to secure nose breathers. If these things can be secured, an A1 nation will quickly follow.

**A Guest-house for Mothers and Babies.**

The National League for Health, Maternity, and Child Welfare, whose influence is potent for good in all movements where the child is concerned, is responsible for a further development in this work. It has organised and financed, and will maintain at Peak Hill Lodge, Sydenham, a guest-house for breast-feeding mothers and their babies who are the wives of unemployed or disabled ex-Service men who may need good air and food but are not definitely ill. A condition of eligibility is that the guests must have attended a local Infant Welfare Centre at least twice before admission. Guests pay their own fares, but no charge whatever is made for their stay, which may extend to four weeks. The Guest-house, whose exterior resembles that of a charming and roomy private country house, was officially opened by the Duchess of Albany on July 13, which was also made a Gift Day, Her Royal Highness receiving the gifts. Such a Guest-house should prove an enormous boon to the mothers, and aids directly in the conservation and improvement of child-life, for which purpose the National League exists. The Marjorie Lumley Holiday Home for Children at Boyne Hill, Maidenhead, which was opened a few months ago, and is maintained by the League, is another home which emphasises the human side of its work.

